# Glutamate: The Master Neurotransmitter and Its Implications in Chronic Stress and Mood Disorders

**DOI:** 10.3389/fnhum.2021.722323

**Published:** 2021-10-29

**Authors:** Mia Michaela Pal

**Affiliations:** In Cognition UK, Church Lawton, United Kingdom

**Keywords:** glutamate, neuroplasticity, GABA, NMDA–receptor, long-term potentiation

## Abstract

This brief review article makes the argument that glutamate is deserving of its newfound attention within the neuroscience literature and that many directions of important research have yet to be explored. Glutamate is an excitatory neurotransmitter with several types of receptors found throughout the central nervous system, and its metabolism is important to maintaining optimal levels within the extracellular space. As such, it is important to memory, cognition, and mood regulation. The mechanisms by which chronic stress affect the glutamatergic system and neuroplasticity are outlined. Several implications for potential pharmacologic and non-pharmacologic interventions are discussed.

## The Sudden Popularity of Glutamate

Until recently, glutamate has often been mentioned only as a sidenote to the more well-known neurotransmitters such as serotonin and norepinephrine. Like the shy kid who suddenly became visible with a new haircut, glutamate has taken the neuroscience literature by storm. This brief review article will explain why glutamate is deserving of this newfound attention and may well be the master neurotransmitter responsible for shaping the entire brain.

## Functions and Mechanisms of Glutamate

### Storage and Transmission

Over the past three decades, researchers have learned that glutamate is the major excitatory neurotransmitter of the healthy mammalian brain, as the most profuse free amino acid that happens to sit at the intersection between several metabolic pathways (Watkins and Jane, 2006; Zhou and Danbolt, 2014). Glutamate is stored in synaptic vesicles of nerve terminals until it is released by exocytosis into the extracellular fluid, where it can quickly become highly concentrated (Zhou and Danbolt, 2014). Additionally, micromolar concentrations of basal extracellular glutamate, originating from non-vesicular release from the cystine-glutamate antiporter, continue to circulate in the space outside the synaptic cleft (Baker et al., 2002). Maintaining optimal levels in this space is essential, as low levels can deplete energy whereas excess levels can lead to cell death (Zhou and Danbolt, 2014). Glutamate transporters located on the outside of astrocytes and neurons quickly act to remove excess glutamate (Zhou and Danbolt, 2014). Receptor proteins at the surface of cells detect glutamate in the extracellular fluid and receive it (Zhou and Danbolt, 2014).

Most cells in the central nervous system (CNS) express at least one type of glutamate receptor. These include the ionotropic *N*-methyl-D-aspartate (NMDA), AMPA (a-amino-3-hydroxy-5-methyl-4-isoxazole propionic acid), and kainite receptors, which mediate fast excitatory transmission; in addition to the family of eight metabotropic glutamate receptors (mGluR1-8), which are located pre-, post-, and extra-syntactically throughout the CNS (Watkins and Jane, 2006; Reznikov et al., 2011; Zhou and Danbolt, 2014). The complex and widespread mechanisms of transmission mean that there is almost unlimited potential for research on each class of receptors and sub-receptors (Watkins and Jane, 2006).

### Neuroplasticity

As a neurostimulator, there is strong support for a role for glutamate in a variety of neuroplasticity mechanisms including long-term potentiation (LTP), regulation of spine density, and synaptic reorganization (Reznikov et al., 2011). As a result, glutamate is now known to be exceptionally important in cognition, learning and mood, all areas in which neuroplasticity is essential to adapting to environmental stressors (Reznikov et al., 2011). LTP in several structures of the CNS employs NMDA and AMPA glutamate receptors to strengthen synaptic connections, necessary for learning and memory (Lynch, 2004; Sah et al., 2008). Morphologic adaptation is necessary for the regulation of mood and cognition (Reznikov et al., 2011).

However, chronic stress can lead to malfunctioning of the glutamate system and reduced neuroplasticity. In the hippocampus, chronic stress leads to increased glutamate release, impaired LTP, atrophy of the apical dendrites, and learning and memory deficits (Reznikov et al., 2011). In the prefrontal cortex, chronic stress leads to decreased glutamate release, impaired LTP, reduced dendritic spines, and impaired attention (Reznikov et al., 2011). In the amygdala, chronic stress leads to decreased glutamate release, impaired or enhanced LTP, dendritic hypertrophy, increased dendritic spines, and anxiety (Reznikov et al., 2011). Guo et al. (2020) have suggested that the negative impact of stress may be due to activation of the microglial cells, which trigger neuroinflammation, affecting both intracellular and extracellular signaling pathways.

## Potential for Future Treatment

### Antidepressant Medications

Glutamate system dysfunction has been implicated in several pre-clinical and clinical studies of mood and disorders. Glutamate reductions have been noted in several neural areas of patients with MDD (Arnone et al., 2015), while mixed results were found with bipolar disorder (Gigante et al., 2012; Chitty et al., 2013), and several glutamatergic genes affecting different kinds of receptors have been implicated in mood disorders (de Sousa et al., 2017). Several glutamatergic agents have been demonstrated to effectively decrease depressive symptoms in people with MDD and bipolar disorder (BD) (Henter et al., 2018).

Among the most studied is ketamine, which rapidly achieves its antidepressant effects with long-lasting effects of a small dose in even treatment resistant MDD and BD (Kantrowitz et al., 2015; Newport et al., 2015; Mandal et al., 2019). Although the mechanisms of ketamine’s actions are still not understood, preclinical studies in mice suggest that found that its antidepressant effects may be produced by the metabolite (2R,6R)-hydroxynorketamine (HNK) that increases AMPA receptor activation (Zanos et al., 2016). Intravenous esketamine, an S(+) enantiomer of ketamine with a high affinity for NMDA receptors, was found to have a rapid and robust antidepressant effect within 2 h in several large randomised controlled trials (RCT) of people with MDD (Singh et al., 2016), and has now been approved within the United States for intranasal administration for people with high risk of suicide (Henter et al., 2018).

Two subunit NR2B-specific NMDA receptor antagonists were recently tested for MDD. While CP-101,606 (traxoprodil) was effective but was halted due to cardiovascular toxicity, MK-0657 (CERC-301) had no significant side effects but had mixed outcomes (Henter et al., 2018). Rapastinel, a glycine partial NMDA agonist, has shown high efficacy in clinical trials for major depression disorder (MDD), and has now been approved for the adjunctive treatment of MDD in the United States (Moskal et al., 2014; Preskorn et al., 2015; Vasilescu et al., 2017). Preliminary results show that sarcosine, a glycine transporter-I inhibitor that potentiates NMDA function, was more effective that citalopram, with no significant side effects (Huang et al., 2013). 4-Cl-KYN (AV-101), a highly selective glycine receptor antagonist, was highly effective in animal studies and is now being tested in clinical trials for MDD (Zanos et al., 2015). Additionally, there are agents that target the mGluRs, but none have been demonstrated to achieve a strong anti-depressive effect (Henter et al., 2018). Thus, the mechanisms and effectiveness of several glutaminergic agents require further study.

### Natural Boosts for Everyday Functioning

Another reason to get glutamate into the public eye is that with minimal knowledge of its mechanisms, there are many natural ways the lay public can boost their overall health and wellbeing. Physical exercise and mindfulness exercises have both been demonstrated to be powerful modulators of non-pharmaceutical glutamate and GABA interventions.

Physical exercise leads to increase levels of both glutamate and GABA (Maddock et al., 2016), resulting in participants feeling energized and focused while also experiencing psychological calm. In adult rats, running has been demonstrated to stimulate neurogenesis and increase the gene expression levels of the NR2B subunit of the NDMA receptor in the dentate gyrus, leading to enhanced learning, memory, and mood functioning (Vivar and van Praag, 2017). In humans, three different experiments show that vigorous physical activity results increased content of glutamate and GABA in the visual and anterior cingulate cortices in comparison with sedentary activity (Maddock et al., 2016). Levels rose approximately five percent and persisted for at least 30 min post-exercise. Additionally, participants who had higher levels of exercise in the previous week also had higher resting glutamate levels.

Mindfulness has a strong impact on brain glutamate levels observed in the brains of people who meditate mindfulness (Fayed et al., 2013). A cross-sectional study comparing the brains of meditators from a Zen Buddhist monastery with hospital staff showed a negative correlation between years of meditation and levels of glutamate in the left thalamus, which may indicate a higher level of efficiency of glutamate metabolism in this area (Fayed et al., 2013). The Zen meditators also had high myo-inositol concentrations in the posterior cingulate, which may indicate higher levels of glial and microglial activation. The exact mechanisms by which glutamate may modulate the effects of mindfulness still must be explored.

## Conclusion

This brief review has highlighted the widespread impact of glutamate throughout the brain health. Glutamate is critical for maintenance of ideal energy levels, necessary for most CNS functions, and neuroplasticity, which is critical for adaptation to changes in the environment. Rather than being delegated as a sidenote as in the past, glutamate is deserving of a main focus in future neuroscience research and clinical studies. Additionally, efforts should be made to educate the lay public as to the importance of glutamate to everyday functioning and how to maintain healthy levels for increased resiliency in times of stress.

## Author Contributions

The author confirms being the sole contributor of this work and has approved it for publication.

## Conflict of Interest

MP is the Director at In Cognition UK, private clinic and sole author to the presented research. This research has been conducted in the absence of any commercial or financial relationship that could be construed as a potential conflict of interest.

## Publisher’s Note

All claims expressed in this article are solely those of the authors and do not necessarily represent those of their affiliated organizations, or those of the publisher, the editors and the reviewers. Any product that may be evaluated in this article, or claim that may be made by its manufacturer, is not guaranteed or endorsed by the publisher.
